# Long noncoding RNA PVT1 promotes chondrocyte extracellular matrix degradation by acting as a sponge for miR-140 in IL-1β-stimulated chondrocytes

**DOI:** 10.1186/s13018-022-03114-4

**Published:** 2022-04-10

**Authors:** Nan Yao, Sha Peng, Huai Wu, Wengang Liu, Dake Cai, Dane Huang

**Affiliations:** 1 Guangdong Provincial Second Hospital of Traditional Chinese Medicine (Guangdong Provincial Engineering Technology Research Institute of Traditional Chinese Medicine), 60 Hengfu Road, Yuexiu District, Guangzhou City, 510095 Guangdong Province China; 2 The Fifth Clinical College of Guangzhou University of Chinese Medicine, 60 Hengfu Road, Yuexiu District, Guangzhou City, 510095 Guangdong Province China; 3 Guangdong Provincial Key Laboratory of Research and Development in Traditional Chinese Medicine, 60 Hengfu Road, Yuexiu District, Guangzhou City, 510095 Guangdong Province China

**Keywords:** Osteoarthritis, Chondrocyte, lncRNA PVT1, miR-140, ceRNA

## Abstract

**Background:**

Osteoarthritis (OA) is a common degenerative joint disease, and chondrocyte extracellular matrix (ECM) degradation is one vital pathological feature of OA. Long noncoding RNA (lncRNA), a new kind of gene regulator, plays an important role in pathogenesis of many diseases like OA. Recent studies have confirmed that lncRNA plasmacytoma variant translocation 1 (PVT1) expression was upregulated in OA patients; however, its effect on ECM degradation remained unknown.

**Methods:**

Cartilage tissue samples were obtained from 6 OA patients admitted in Guangdong Second Traditional Chinese Medicine Hospital. Chondrocytes were isolated and cultured from the collected cartilage tissue. Plasmid construction, RNA interference, cell transfection, fluorescence in situ hybridization (FISH), and pull-down assay were carried out during the research.

**Results:**

In this study, PVT1 expression was significantly increased in chondrocytes stimulated by interleukin-1β (IL-1β). In addition, inhibition of PVT1 significantly downregulated the increased expressions of ADAM metallopeptidase with thrombospondin type 1 motif-5 (ADAMTS-5) and matrix metalloproteinase-13 (MMP-13) induced by IL-1β. Further investigation revealed that PVT1 was an endogenous sponge RNA, which directly bound to miR-140 and inhibited miR-140 expression.

**Conclusion:**

To sum up, this study showed that PVT1 promoted expressions of ADAMTS-5 and MMP-13 as a competing endogenous RNA (ceRNA) of miR-140 in OA, which eventually led to aggravation of ECM degradation, thus providing a new and promising strategy for the treatment of OA.

## Background

Osteoarthritis (OA) is a whole joint disease involved in articular cartilage, subchondral bone, meniscus, synovial membrane, and infrapatellar fat pad [1]. Articular cartilage is mainly composed of chondrocytes and extracellular matrix (ECM), and chondrocytes secrete substances that participate in the anabolism or catabolism of ECM. In generally, the anabolism or catabolism of ECM is in dynamic balance, thus maintaining a balanced microenvironment in joint. Once the ECM metabolism is imbalance, the consequent ECM degradation will promote cartilage degeneration and accelerate the development of OA. OA is a chronic inflammatory disease, influenced by a vital inflammatory cytokine named interleukin-1β (IL-1β). In the process of ECM degradation, IL-1β regulates expressions of ADAM metallopeptidase with thrombospondin type 1 motif-5 (ADAMTS-5) of ADAMTS family and matrix metalloproteinase-13 (MMP-13) of MMPs family, thereby aggravating ECM degradation [2]. At present, the mechanism of cartilage degeneration has not been well elucidated, and anti-OA drugs on the market only relieve pain rather than reversing the course of OA [3]. Therefore, it is of great significance to explore the deep molecular mechanism of cartilage degeneration. The expression level changes of microRNA (miRNA) may contribute to abnormal expressions of some genes and trigger the outset of OA. For example, miR-140 is a recognized miRNA that plays an important role in the pathogenesis of OA, and its role in maintaining the balanced state of cartilage ECM is particularly obvious [4, 5]. It has been reported that ADAMTS-5 and MMP-13 are two downstream target genes of miR-140. The miR-140 expression is markedly decreased, inducing the aggravation of ECM degradation caused by ADAMTS-5 and MMP-13 in OA [6].

In addition, plasmacytoma variant translocation 1 (PVT1) is a kind of lncRNA located at 8q24.21, which has been proved to play a regulatory role in many cancers and may become a potential biomarker of clinical pathological characteristics of different types of cancers [7]. And a recent study found that PVT1 expression in OA cartilage tissues is almost three times higher compared with normal cartilage tissues in OA [8]. We predicted that PVT1 might act as an important regulator in the pathogenesis of OA. There is more and more evidence showing that lncRNA PVT1 exerts a sponge-like miRNA in chondrocytes [9–12]. However, whether PVT1 adsorbs miR-140 as a competing endogenous RNA (ceRNA) and regulates ECM metabolism in chondrocytes remains unknown. According to the prediction of starBase2.0, we found that PVT1 and miR-140 have binding sites. Therefore, the purpose of this study is to analyze the interaction between PVT1 and miR-140 in chondrocytes and their effects on chondrocyte ECM metabolism.

## Materials and methods

### Isolation and culture of human primary chondrocytes

Cartilage tissue samples were obtained from 6 patients (3 males and 3 females, age 65.8 ± 6.5 years) with OA undergoing total joint replacement surgery in the Orthopedic Department of Guangdong Second Traditional Chinese Medicine Hospital. OA was confirmed using radiographic images and diagnosed according to the American College of Rheumatology criteria. Patients with rheumatoid arthritis, gouty arthritis, traumatic arthritis, and other knee diseases were excluded. All the patients provided informed consent to be part of the current study, and the surgery and cartilage collection was approved by the Ethical Committee of Guangdong Second Traditional Chinese Medicine Hospital (Approval no. 2019-76). As previously reported, chondrocytes were isolated and cultured from the collected cartilage tissue after the corresponding treatment [13]. To prepare the primary chondrocytes, the collected cartilage was minced and pretreated with 0.25% trypsin (Gibco, Rockville, MD, USA) for 30 min. Then, the tissue slices were digested over night with 0.2% collagenase II (Invitrogen, Carlsbad, CA, USA) in Dulbecco’s modified Eagle’s medium (DMEM; Gibco, Rockville, MD, USA) containing 10% fetal bovine serum (FBS; Bovogen, Melbourne, Australia). The isolated cells were then passed through a filter to remove the residual cartilage matrix fragments, followed by centrifugation. Afterward, cells were resuspended in DMEM medium supplemented with 10% FBS and 1% Pen Strep (Gibco, Rockville, MD, USA). All cells were cultured in a humidified atmosphere with 5% CO_2_ at 37 °C. In addition, chondrocytes were subjected to the stimulation of IL-1β (10 ng/mL; PeproTech, Rocky Hill, NJ, USA) for 24 h as an OA cell model.

### Reverse transcription-quantitative polymerase chain reaction (RT-qPCR)

Total RNA from chondrocytes was extracted using the TRIzol reagent (Thermo Fisher Scientific, Waltham, MA, USA). Then, reverse transcription was performed to synthesize first-strand cDNA by using PrimeScript™ RT Master Mix (Takara, Dalian, China) from each RNA sample (1 μg). Afterward, the obtained cDNA was subjected to real-time PCR to evaluate the gene expression levels, including lncRNA-PVT1, miR-140, ADAMTS-5, and MMP-13. All PCR reactions were performed by using TB Green™ Premix Ex Taq™ II (Tli RNaseH Plus) (2×) (Takara, Dalian, China) on the StepOnePlus Real-Time PCR system (Thermo Fisher Scientific, Waltham, MA, USA). The specific primers (Thermo Fisher Scientific, Waltham, MA, USA) used in the study are displayed in Table 1. For normalization, U6 was used as an internal reference for miR-140, and GAPDH was used as an internal reference for others. The relative gene expressions were calculated according to the 2^−△△Ct^ method.

**Table 1:** Information on primer sequences

Gene	Sequences (5′–3′)
PVT1	Forward primer: CTGAAGGTTCCTAAGCCTCTAAG Reverse primer: AGTGGTTTTTCCATTATTGGTATT
miR-140	Forward primer: CCCCCAGTGGTTTTACCCTA Reverse primer: GTGCGTGTCGTGGAGTCG
ADAMTS-5	Forward primer: GATGGCACTGAATGTAG Reverse primer: ACTCCGCACTTGTC
MMP-13	Forward primer: ATGCATCCAGGGGTCCTGGC Reverse primer: TGCTGCATTCTCCTTCAGGA
U6	Forward primer: GCTTCGGCAGCACATATACTAAAAT Reverse primer: CGCTTCACGAATTTGCGTGTCAT
GAPDH	Forward primer: CCCATCACCATCTTCCAGGAG Reverse primer: CTTCTCCATGGTGGTGAAGACG

*PVT1* plasmacytoma variant translocation 1, *miR-140* microRNA-140, *ADAMTS-5* ADAM metallopeptidase with thrombospondin type 1 motif-5, *MMP-13* matrix metalloproteinase-13, *GAPDH* glyceraldehyde-3-phosphate dehydrogenase

### Western blot

Total protein from chondrocytes was homogenized and lysed with RIPA solution and protease phosphatase inhibitor mixture (BestBio, Shanghai, China). The concentration of extracted protein was detected by a BCA kit (Thermo Fisher Scientific, Waltham, MA, USA). Then, each protein sample (30 µg) was loaded into 10% SDS-PAGE gel and then was transferred onto PVDF membranes (Millipore, Billerica, MA, USA). After incubation with 5% non-fat milk for 1.5 h to interdict non-specific binding, the membranes were then incubated with primary antibodies overnight at 4 °C. Primary antibodies were shown as follows: ADAMTS-5 (ab41037, 1:1000; Abcam, Cambridge, MA, USA), MMP-13 (ab39012, 1:1000; Abcam, Cambridge, MA, USA), and GAPDH (ab8245, 1:1000; Abcam, Cambridge, MA, USA). The membranes were then incubated with HRP-labeled secondary antibodies at room temperature for 1.5 h. One and a half hour later, the binding signals were visualized by SuperSignal West Pico PLUS Chemiluminescent Substrate (Thermo Fisher Scientific, Waltham, MA, USA). The densitometric assay of bands was captured using an imaging system (Tanon, Shanghai, China), and the relative protein expressions were analyzed with Image J software. For normalization, GAPDH was used as an internal reference.

### Plasmid construction, RNA interference, and transfection

The full-length lncRNA PVT1 was introduced into the pcDNA3.1(+) vector (Invitrogen, Carlsbad, CA, USA) and was designated as pcDNA-PVT1 plasmid. Small interfering RNA (siRNA) against PVT1 (si-PVT1) was designed and synthesized by GenePharma (Shanghai, China). Oligonucleotide sequences of miR-140 inhibitor, negative control (NC) inhibitor, miR-140 mimics, and NC mimics were also obtained from GenePharma (Shanghai, China). When the cells reached 80% confluence, the recombinant plasmids or oligonucleotides were transfected into cells using Lipofectamine 2000 (Thermo Fisher Scientific, Waltham, MA, USA) according to the manufacturer's instructions. The chondrocytes were harvested 48 h after transfection for further experiments.

### Luciferase reporter assay

The short sequence of wild-type miR-140-binding bites on PVT1 (PVT1-wt) and the mutated short sequence lacking of the miR-140-binding bites on PVT1 (PVT1-mut) were synthesized and inserted into pSi-Check2 vector (Promega, Madison, WI, USA). Chondrocytes were co-transfected with pSi-Check2 containing PVT1-wt or PVT1-mut, and miR-140 mimics or NC mimics using Lipofectamine 2000. Luciferase activity was measured by using a Dual-Luciferase Reporter Assay System (Promega, Madison, WI, USA).

### Fluorescence in situ hybridization (FISH)

DNA oligo probes (GenePharma, Shanghai, China) labeled with FAM for PVT1 and Cy3 for miR-140 were utilized in FISH assays, whereas the cell nuclei were counterstained with 4,6-diamidino-2-phenylindole (DAPI; Servicebio, Wuhan, China). All procedures were carried out according to the manufacturer’s instructions, and all images were acquired using a DMI8 microscope (Leica Microsystems, Mannheim, Germany).

### Pull-down assay with biotinylated RNA probe

For PVT1 expression detection, chondrocytes were transfected with biotinylated miR-140 mimics (GenePharma, Shanghai, China) for 48 h. After rinsing with cold PBS, cells were lysed with lysis buffer and incubated with magnetic beads. Three hours later, the samples were washed and the bound RNAs were then extracted and subjected into RT-qPCR assay to detect PVT1 expression. For miR-140 expression detection, biotinylated PVT1 probe (GenePharma, Shanghai, China) was processed through the same protocol.

### Statistical analysis

All data were analyzed by software SPSS 22.0 (SPSS, Chicago, IL, USA), and the data were expressed as mean ± standard deviation. One-way analysis of variance was applied to analyze differences in data of all parameters among the different groups. SNK method was used when it had equal variance, while Dunnett’s T3 method was used when it had unequal variance. Differences were considered statistically significant when *P* < 0.05.

## Results

### Effect of IL-1β on the gene expressions in chondrocytes

**Fig. 1:**
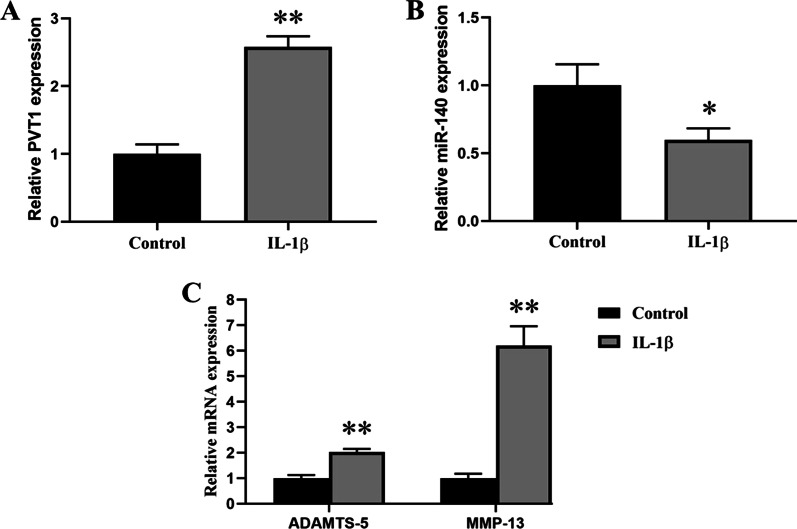
Effect of IL-1β on the gene expressions in chondrocytes. **A** Comparison of PVT1 expression between control and IL-1β-stimulated chondrocytes. **B** Comparison of miR-140 expression between control and IL-1β-stimulated chondrocytes. **C** Comparison of ADAMTS-5 and MMP-13 expression between control and IL-1β-stimulated chondrocytes. The results were represented as mean ± SD. *P < 0.05; **P < 0.01 vs control

In order to establish OA cell model in vitro, chondrocytes were treated with 10 ng/mL of IL‑1β. The gene expressions of PVT1, miR-140, ADAMTS-5, and MMP-13 in chondrocytes were analyzed by RT-qPCR. Compared with control chondrocytes, PVT1 expression was significantly increased in IL‑1β-stimulated chondrocytes (Fig. 1A). In addition, miR-140 expression was remarkably downregulated in IL‑1β-stimulated chondrocytes (Fig. 1B), and the mRNA expressions of ADAMTS-5 and MMP‑13 were significantly increased in IL‑1β-stimulated chondrocytes (Fig. 1C).

### Effect of lncRNA-PVT1 on the gene expressions in chondrocytes

**Fig. 2:**
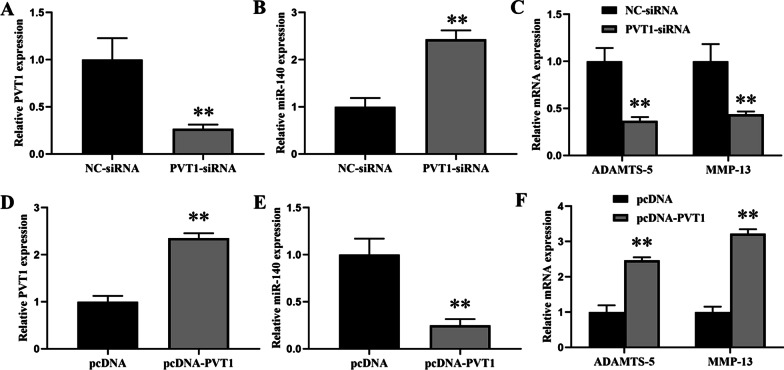
Effect of lncRNA PVT1 on the gene expressions in chondrocytes. **A** Relative PVT1 expression was measured in chondrocytes transfected with NC-siRNA or PVT1-siRNA. **B** Relative miR-140 expression was measured in chondrocytes transfected with NC-siRNA or PVT1-siRNA. **C** Relative mRNA expression was measured in chondrocytes transfected with NC-siRNA or PVT1-siRNA. **D** Relative PVT1 expression was measured in chondrocytes transfected with pcDNA or pcDNA-PVT1. **E** Relative miR-140 expression was measured in chondrocytes transfected with pcDNA or pcDNA-PVT1. **F** Relative mRNA expression was measured in chondrocytes transfected with pcDNA or pcDNA-PVT1. The results were represented as mean ± SD. **P < 0.01 vs NC-siRNA (**A**–**C**), **P < 0.01 vs pcDNA (**D**–**F**)

To investigate the role of lncRNA PVT1 in chondrocytes, we not only designed a pcDNA-PVT1 plasmid to increase PVT1 expression, but also designed a siRNA to specifically suppress PVT1 expression. Then, we observed the effect of pcDNA-PVT1 or siRNA-PVT1 on relative gene expressions. PVT1-siRNA significantly suppressed the PVT1 expression and increased the expression of miR-140, concomitantly with downregulation of ADAMTS-5 and MMP-13 mRNA expressions (Fig. 2A–C). However, overexpression of PVT1 by transfection of pcDNA-PVT1 plasmid significantly increased the PVT1 expression and remarkably downregulated the expression of miR-140 accompanied with an overt upregulation of ADAMTS-5 and MMP-13 mRNA expressions (Fig. 2D–F). Overall, these data suggested that lncRNA PVT1 was involved in the ECM degradation in chondrocytes.

### Effect of interaction between lncRNA PVT1 and miR-140 in chondrocytes

**Fig. 3:**
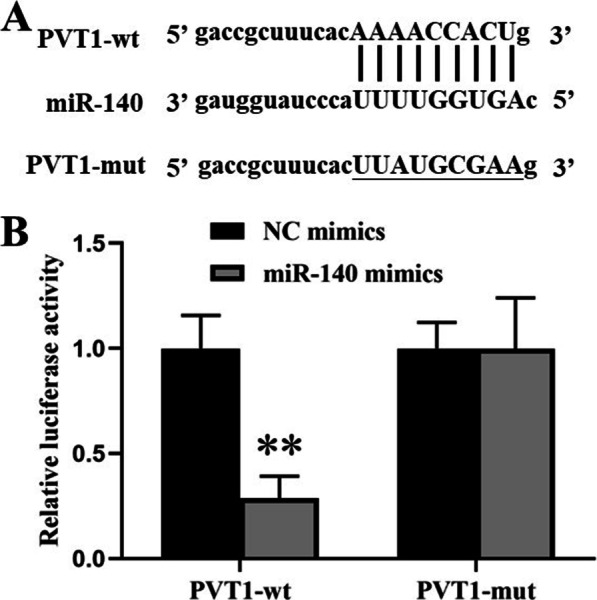
**A** Bioinformatics analysis of matching sequence of miR-140 within 3’-UTR of PVT1. **B** Luciferase activity was measured in chondrocytes co-transfected with NC mimics or miR-140 mimics and PVT1-wt or PVT1-mut at 48 h after transfection. The results were represented as mean ± SD. **P < 0.01 vs NC mimics

**Fig. 4:**
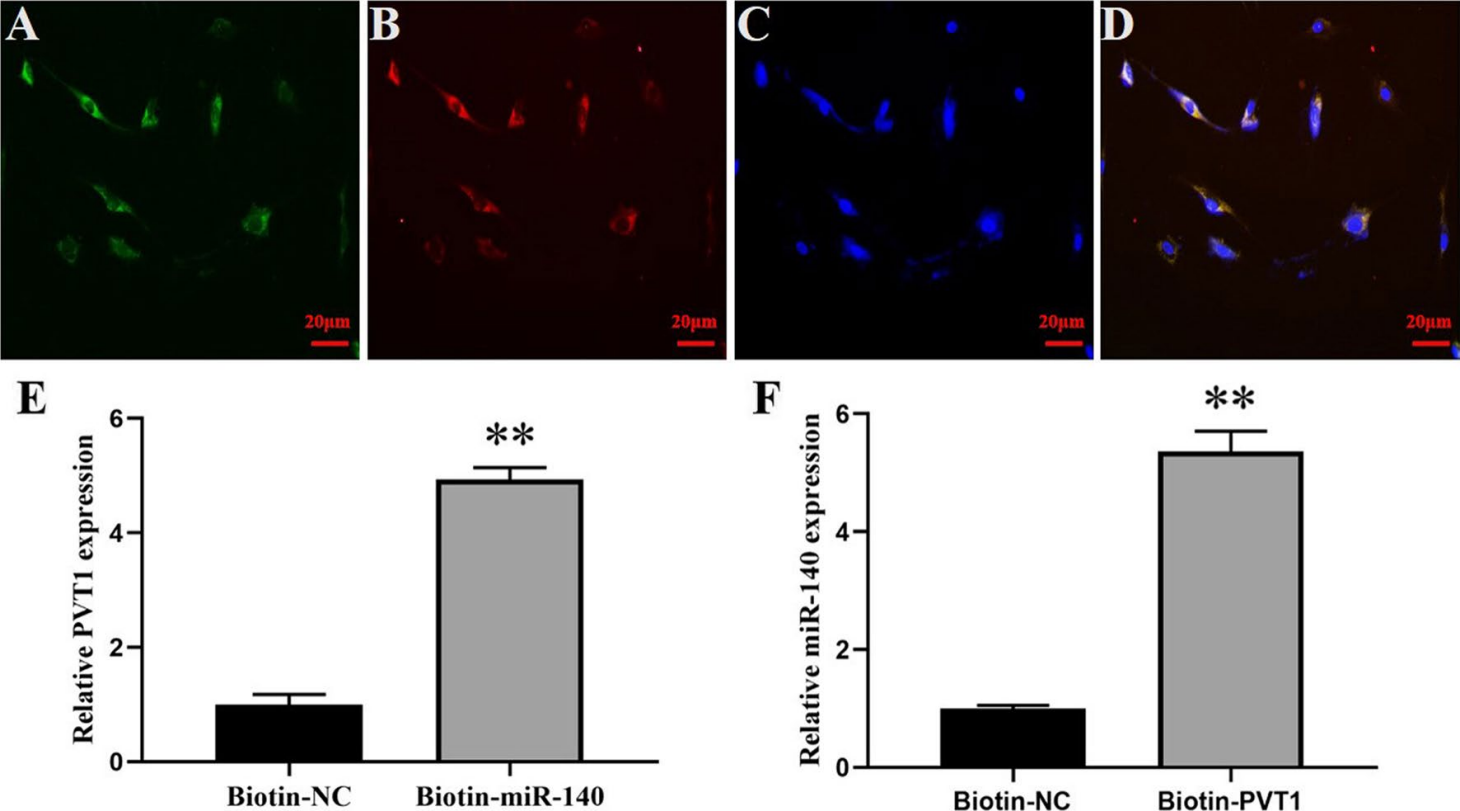
**A**–**D** PVT1 and miR-140 were co-localized in chondrocytes by FISH. **A** PVT1 was stained green; **B** miR-140 was stained red; **C** cell nuclei were stained blue; **D** overlapped expression was mixed (scale bar, 20 μm). **E** Relative lncRNA PVT1 expression was detected in Biotin-miR-140 group compared with Biotin-NC group. **F** Relative miR-140 expression was detected in Biotin-PVT1 group compared with Biotin-NC group. The results were represented as mean ± SD. **P < 0.01 vs Biotin-NC (**E**, **F**)

We performed bioinformatics analysis (starBase 2.0) to predict the potential binding sites for PVT1 and miR-140 (Fig. 3A). To confirm the interaction of PVT1 with miR-140, luciferase report genes were constructed. Luciferase activity assays showed that miR-140 mimics led to a notable decrease in luciferase activity in PVT1-wt group compared with that of NC mimics group. However, no significant effect of luciferase activity was observed in PVT1-mut group compared with that of NC mimics group (Fig. 3B). In order to further identify the interaction between PVT1 and miR-140 in chondrocytes, we next carried out FISH and RNA pull-down assays. FISH assay demonstrated that PVT1 expressed by green fluorescence and miR-140 expressed by red fluorescence could be visualized in chondrocytes, and co-localization was observed in chondrocytes (Fig. 4A–D). RNA pull-down assay showed that PVT1 expression was significantly elevated in the Biotin-miR-140 group compared with that of Biotin-NC group. Moreover, miR-140 expression was also significantly elevated in Biotin-PVT1 group compared with that of Biotin-NC group (Fig. 4E, F). All these results revealed that PVT1 directly bound to miR-140.

### Effect of PVT1 on ADAMTS-5 and MMP-13 expressions by acting as a ceRNA of miR-140 in IL-1β-stimulated chondrocytes

**Fig. 5:**
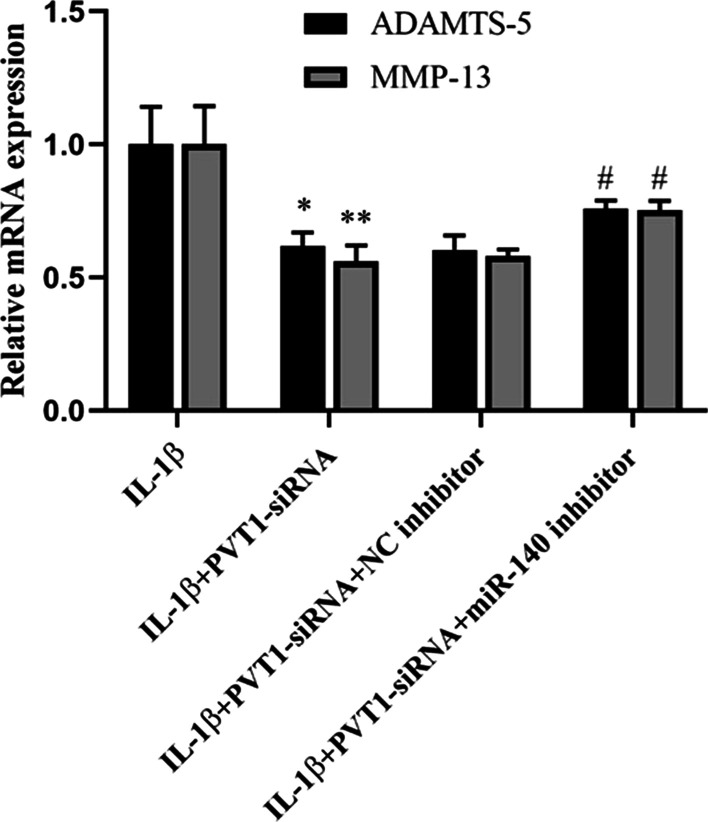
Effect of PVT1 on ADAMTS-5 and MMP-13 mRNA expressions by acting as a ceRNA of miR-140 in IL-1β-stimulated chondrocytes. The results were represented as mean ± SD. *P < 0.05, **P < 0.01 vs IL-1β. ^#^*P* < 0.05 vs IL-1β + PVT1-siRNA

**Fig. 6:**
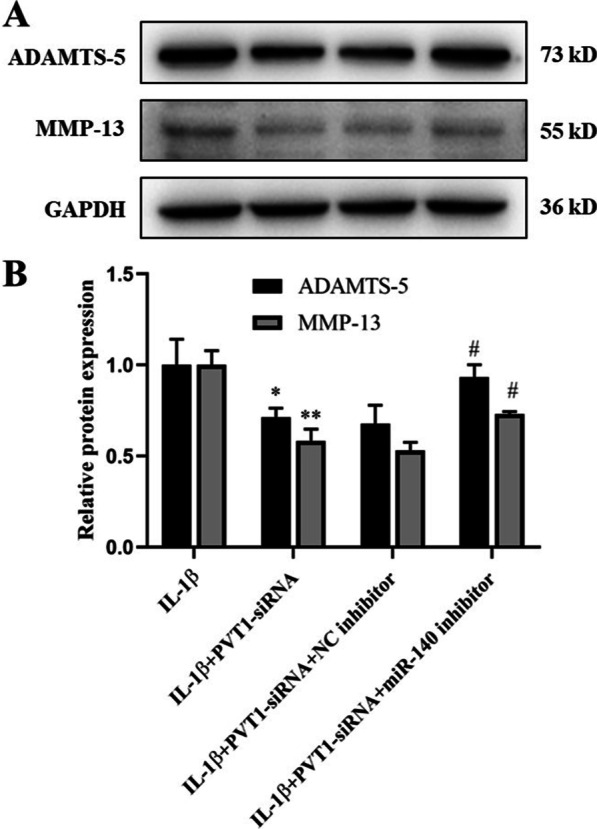
**A** Western blot analysis of ADAMTS-5, MMP-13, and GAPDH. **B** Effect of PVT1 on ADAMTS-5 and MMP-13 protein expressions by acting as a ceRNA of miR-140 in IL-1β-stimulated chondrocytes. The results were represented as mean ± SD. *P < 0.05, **P < 0.01 vs IL-1β. ^#^*P* < 0.05 vs IL-1β + PVT1-siRNA

To prove whether the effect of PVT1 on ECM degradation through a ceRNA mechanism containing miR-140 in IL-1β-stimulated chondrocytes, we investigate the role of miR-140 in PVT1 silence-mediated conditions. The mRNA and protein expressions of ADAMTS-5 and MMP-13 were inhibited in IL-1β-stimulated chondrocytes after transfection with PVT1-siRNA (Fig. 5 and Fig. 6A, B). Furthermore, miR-140 suppression antagonized the inhibitory effect of PVT1 silence on ADAMTS-5 and MMP-13 expressions (Figs. 5, 6A, B).

## Discussion

As we know, the common pathological manifestation of OA is articular cartilage degeneration. In the pathogenesis of OA, the ECM degradation is closely related to ADAMTS and MMPs family. The ADAMTS family contains 19 secretory multidomain proteolytic enzymes, among which ADAMTS-5 is the most important proteoglycan enzyme and plays a decisive role in the degradation of proteoglycan in chondrocyte ECM [14, 15]. The MMPs family is a kind of protease superfamily which widely exists in various tissues and plays a vital part in the degradation of type II collagen [16]. Notably, MMP-13 is the most active type II collagen lyase, which mainly degrades type II collagen in the pathogenesis of OA [17]. In a word, these two enzymes are involved in ECM degradation in the pathogenesis of OA. Inflammatory factors are crucial pathogenic factors in the pathogenesis of OA, among which IL-1β is closely related to the pathogenesis of OA and is the main cytokine that leads to promotion of ECM degradation, imbalance of articular cartilage metabolism, and articular cartilage injury [18–21]. In this study, ADAMTS-5 and MMP-13 are considered as markers of ECM degradation.

LncRNA is a kind of noncoding protein single-stranded RNA with a length of more than 200 nucleotides, which interferes with various functions of cells [22]. With the extensive development of noncoding RNA in OA research, the regulatory role of lncRNA has gradually been paid attention to in the pathogenesis of OA [23]. At first, lncRNA PVT1 was discovered as an activator of MYC in mouse plasmacytoma translocation, and later it was found that lncRNA PVT1 played a carcinogenic part in various human cancers [24, 25]. Additionally, PVT1 expression is upregulated in OA patients and chondrocytes stimulated by IL-1β [8, 11, 12]. MiRNA is a kind of noncoding protein single-stranded small RNA with a length of about 18–22 nucleotides encoded by endogenous genes, which usually affects the transcription and translation of target genes, and then affects the function of cells [26]. At present, research shows that miRNAs influence the pathological process of OA [27]. Among them, miR-140 is a specific miRNA with high expression in chondrocytes, which is primarily responsible for maintaining normal cartilage metabolism, that is, maintaining chondrocyte ECM homeostasis [28]. In chondrocytes, miR-140 can inhibit the expression of ADAMTS-5 and MMP-13, and it has been proved that ADAMTS-5 and MMP-13 are the target genes of miR-140 [5, 29].

In this study, low expression of miR-140 and high expression of PVT1, ADAMTS-5, and MMP-13 were found in IL-1β-stimulated chondrocytes. Therefore, we speculated that close relationship was emerged between PVT1 and miR-140. Further study found that silencing PVT1 expression by siRNA increased miR-140 expression with decrease expressions of ADAMTS-5 and MMP-13. Furthermore, overexpression of PVT1 inhibited miR-140 expression, which led to increased expressions of ADAMTS-5 and MMP-13. At present, more and more studies support that lncRNA can be used as ceRNA, which can adsorb miRNA in the form of natural sponge scaffold and exert effects on the pathogenesis of OA [11, 12]. In this study, starBase 2.0 predicted that miR-140 could be the target of PVT1, indicating that PVT1 might be a ceRNA of miR-140. Subsequently, co-localization and luciferase detection confirmed that PVT1 was a direct target of miR-140. Furthermore, antisense experiment confirmed that blocking miR-140 expression in chondrocytes stimulated by IL-1β could reverse the decrease expressions of ADAMTS-5 and MMP-13 caused by PVT1 inhibition.

## Conclusion

In conclusion, this study not only confirmed the increase expression of PVT1 in chondrocytes stimulated by IL-1β, but also showed that PVT1 silencing promoted the expression of miR-140 by acting as a sponge of miR-140, and resisted the metabolic imbalance caused by IL-1β exposure. LncRNA-PVT1 was found as a sponge of miR-140 in chondrocytes for the first time, which regulated the expression of ADAMTS-5 and MMP-13 and participated in the ECM degradation in OA. Taken together, this study clarifies how PVT1 aggravates the development of OA, and supports PVT1 as a potential target to treat OA.

## Data Availability

The datasets used and/or analyzed during the current study are available from the corresponding author on reasonable request.
